# OUTCOME OF LAPAROSCOPIC CHOLECYSTECTOMY IN PATIENTS WITH GALLSTONE DISEASE AT A SECONDARY LEVEL CARE HOSPITAL

**DOI:** 10.1590/0102-672020180001e1347

**Published:** 2018-06-21

**Authors:** Ahmed TAKI-ELDIN, Abd-Elnaser BADAWY

**Affiliations:** 1 General Surgery Department; 2 Biochemistry Department, Faculty of Medicine, Northern Border University, Arar, KSA (Saudi Arabia)

**Keywords:** Cholecystectomy, Laparoscopy, Cholelithiasis, Complications.., Colecistectomia, Laparoscpia, Colelitíase, Complicações

## Abstract

**Background:**

Laparoscopic cholecystectomy is the most commonly performed operation of the digestive tract. ^)^It is considered as the gold standard treatment for cholelithiasis.

**Aim:**

To evaluate the outcome of it regarding length of hospital stay, complications, morbidity and mortality at a secondary hospital.

**Methods:**

Data of 492 patients who underwent laparoscopic cholecystectomy were retrospectively reviewed. Patients’ demographics, co-morbid diseases, previous abdominal surgery, conversion to open cholecystectomy, operative time, intra and postoperative complications, and hospital stay were collected and analyzed from patients’ files.

**Results:**

Out of 492 patients, 386 (78.5%) were females and 106 (21.5%) males. The mean age of the patients was 49.35±8.68 years. Mean operative time was 65.94±11.52 min. Twenty-four cases (4.9%) were converted to open surgery, four due to obscure anatomy (0.8%), 11 due to difficult dissection in Calot’s triangle (2.2%) and nine by bleeding (1.8%). Twelve (2.4%) cases had biliary leakage, seven (1.4%) due to partial tear in common bile duct, the other five due to slipped cystic duct stables. Mean hospital stay was 2.6±1.5 days. Twenty-one (4.3%) developed wound infection. Port site hernia was detected in nine (1.8%) patients. There was no cases of bowel injury or spilled gallstones. There was no mortality recorded in this series.

**Conclusions:**

Laparoscopic cholecystectomy is a safe and effective line for management of gallstone disease that can be performed with acceptable morbidity at a secondary hospital.

## RESUMO

**Racional:**

A colecistectomia laparoscópica é a operação mais comum do aparelho digestivo. É considerada como o tratamento padrão-ouro para colecistolitíase.

**Objetivo:**

Avaliar o resultado dela quanto ao tempo de internação, complicações, morbidade e mortalidade em um hospital secundário.

**Métodos:**

Foram analisados ​​retrospectivamente dados de 492 doentes submetidos à colecistectomia laparoscópica. Os dados demográficos, as comorbidades, operação abdominal prévia, conversão para colecistectomia laparotômica, tempo cirúrgico, complicações intra e pós-operatórias e internação hospitalar foram coletados e analisados ​​a partir dos prontuários.

**Resultados:**

Dos 492 pacientes, 386 (78,5%) eram mulheres e 106 (21,5%) homens. A idade média foi de 49,35±8,68 anos. O tempo operatório médio foi de 65,94±11,52 min. Vinte e quatro casos (4,9%) foram convertidos em laparotomia quatro devido à anatomia obscura (0,8%), 11 por dissecção difícil no triângulo de Calot (2,2%) e nove por sangramento (1,8%). Doze (2,4%) casos apresentaram vazamento biliar, sendo sete (1,4%) devido a ruptura parcial do ducto biliar comum, e os outros cinco por soltura da clipagem do ducto cístico. A média de internação foi de 2,6±1,5 dias. Vinte e um (4,3%) pacientes desenvolveram infecção da ferida. Hérnia do local dos portais foi detectada em nove (1,8%) pacientes. Não houve casos de lesão intestinal ou cálculos biliares soltos na cavidade. Não houve mortalidade

**Conclusões:**

Colecistectomia laparoscópica é operação segura e eficaz no tratamento da colecistolitíase e pode ser realizada com morbidade aceitável em hospitais secundários.

## INTRODUCTION

Laparoscopic cholecystectomy (LC) represents a significant change in the management of gallbladder disease and it is the most commonly performed operation of the digestive tract^8^. It is considered as the gold standard treatment for cholelithiasis. It replaced open cholecystectomy as the first choice of treatment for gallstones and inflammation of the gallbladder. It was made for the first time in 1987 by Muret. Despite many modified methods (natural orifice transluminal endoscopic surgery - NOTES -, single-incision laparoscopic surgery^3^), LC is still the gold standard for symptomatic gallstone disease^4^.

The advantages of the laparoscopic approach are less postoperative pain, shorter hospital stay, faster recovery, improved cosmetic results, early return to work, fewer complications such as infection^11^, adhesions, short operating time and low learning curve^7^ ^,^ ^10^ ^,^ ^16^ and, it is superior to other developed techniques because of economic advantage^10^.

The main risk associated with the LC appears to be a higher incidence of bile duct injury than open cholecystectomy, 0.3-0.8%^9^ ^,^ ^19^.

 In addition, there are also risks to other complications such as major vascular injuries, injury to intestine, clips migration, biliary leak, cautery injuries and complications from lost gallstones^1^ ^,^ ^14^ ^,^ ^15^ ^,^ ^20^.

The outcome of laparoscopic cholecystectomy in literature has been assessed by many different outcomes measures: bile duct injuries, conversion rates, morbidity and mortality. However, there remains considerable debate which measures should be used to reflect surgical quality, as the various measures have strengths and weaknesses^18^.

The aim of this study was to evaluate the outcome of LC in context to its complications, morbidity and mortality in a secondary care hospital. 

## METHODS

This retrospective study was conducted on 492 patients, who underwent LC, during the period from January 2011 to December 2015, at General Surgery Department, Arar Central Hospital, Northern Borders Region, Saudi Arabia**.** A written and informed consent was taken from all the patients included in the study before LC. The protocol of the study was approved by the local ethical committee.

The following data were reviewed; patient’s demographics (age, gender), preoperative investigations (routine in all patients CBC, bleeding-coagulation times, liver function tests, renal function tests, blood glucose level, screening for hepatitis), systemic diseases (diabetes mellitus, hypertension, liver, renal, lung diseases, obesity and cardiac problems). American Society of Anesthesiologist’s (ASA) score, indication for surgery (elective/emergency), intra-operative findings (duration of operation, intra-operative bleeding and iatrogenic injuries), conversion from laparoscopic to open cholecystectomy and reason for conversion, postoperative complications, early (hemorrhage, bile leak, wound infection) and late complications (biliary stricture and port site hernia), hospital stay and mortality were reviewed from patients’ records. Patients having incomplete data on their files were not included in the study.

Ultrasonography was routinely performed on all patients to confirm the clinical diagnosis of cholelithiasis with number of stones, sizes, gall-bladder wall thickness, pericholecystic collection, and diameter of common bile duct.

LC was performed using the standard four port technique and pneumoperitoneum was created using Hasson or Veress needle technique. During this study period, five consultant surgeons with vast experience in the field of laparoscopy performed LC.

After surgery, patients were followed up after one week, one month and three months.

### Statistical analysis 

Was performed with the use of SPSS (version 20.0). Demographic characteristics were expressed in frequency and percentage. All the continuous variables were assessed for the normality. If variables were normally distributed, they were expressed as mean±standard deviation, otherwise as median. The qualitative data were analyzed using the Chisquare test and quantitative data by the Student *t*test. P value <0.05 was considered statistically significant.

## RESULTS

A total of 492 patients underwent LC during this study period; out of them, 386 (78.5%) were females and 106 (21.5%) males (female/male ratio of 3.6:1). The mean age of the patients was 49.35±8.68 years (26-72, Table 1).

**TABLE 1:** Demographic and preoperative patient characteristics

Characteristics	Year					Total(n=492) n (%)
	2011 (n=84) n(%)	2012 (n=92) n(%)	2013 (n=106) n(%)	2014 (n= 98) n(%)	2015 (n=112) n (%)	
Gender						
Female	65(77.4%)	68(73.9%)	81(76.4%)	74(75.5%)	98(87.5%)	386(78.5%)
Male	19(22.6%)	24(26.1%)	25(23.6%)	24(24.5%)	14(12.5%)	106(21.5%)
Age						
<40	11(13%)	9(9.8%)	14(13.2%)	17(17.3%)	13(11.6%)	64(13%)
40 - 49	37(44%)	51(55.4%)	40(37.7%)	38(38.8%)	57(50.9%)	223(45.3%)
50 - 59	22(26.2%)	24(26.1%)	42(39.6%)	19(19.4%)	30(26.8%)	137(27.8%)
≥60	14(16.7%)	8(8.7%)	10(9.4%)	24(24.4%)	12(10.7%)	68(13.8%)
Comorbidity						
Diabetes mellitus	35(41.7%)	42(45.7%)	46(43.4%)	43(43.9%)	39(34.8%)	205(41.7%)
Hypertension	30(35.7%)	36(39.1%)	32(30.2%)	26(26.5%)	42(37.5%)	166(33.7%)
Coronary diseases	8(9.5%)	6(6.5%)	12(11.3%)	8(8.2%)	8(7.1%)	42(8.5%)
Valvular diseases	3(3.6%)	4(4.3%)	3(2.8%)	7(7.1%)	2(1.8%)	19(3.9%)
Respiratory diseases	13(15.5%)	11(12%)	14(13.2%)	7(7.1%)	12(10.7%)	57(11.6%)
Obesity	26(31%)	42(45.7%)	58(54.7%)	62(63.3%)	46(41.1%)	234(47.6%)
Previous abdominal surgery	9(10.7%)	10(10.9%)	7(6.6%)	8(8.2%)	19(17 %)	53(10.8%)
Indication of surgery						
Chronic calculous cholecystitis	78(92.9%)	78(84.8%)	94(88.7%)	82(83.7%)	92(82.1%)	424(86.2%)
Chronic acalculous cholecystitis	0(0%)	5(5.4%)	4(3.8%)	4(4.1%)	6(5.4%)	19(3.9%)
Acute cholecystitis	4(4.8%)	10(10.9%)	8(7.5%)	10(10.2%)	12(10.7%)	44(8.9%)
Gall bladder polyp	2(2.4%)	0(0%)	0(0%)	1(1.0%)	2(1.8%)	5(1%)

History of hypertension was found in 166 (33.7%) patients, diabetes mellitus in 205 (41.7%), respiratory diseases (bronchial asthma and COPD) in 57 (11.6%), coronary diseases (angina pectoris and myocardial infarction) in 42 (8.5%), valvular diseases in 19 (3.9%) patients and obesity in 234 (47.6%). History of previous abdominal surgery was found in 53 (10.8 %) patients (Table 1).

The indications of surgery were symptomatic chronic calculous cholecystitis in 424 patients (86.2%), chronic acalculous cholecystitis in 19 (3.9%), acute cholecystitis in 44 (8.9%), and gall bladder polyp in five (1%) patients (Table 1). Patients with calculous obstructive jaundice were referred to higher centers and were not included in this study.

 According to American Society of Anaesthesiologists (ASA) classification, 114 (23.2%) patients were ASA I, 335 (68%) were ASA II, and 43 (8.7%) were ASA III. 

The mean operative time was 65.94±11.52 (range: 45-100 min) and intra-abdominal drain was placed in 128 (26%) patients.

Conversion from laparoscopic to open cholecystectomy was performed in 24 cases (4.9%). The causes for conversion to open cholecystectomy were obscure anatomy in four (0.8%) cases, difficult dissection in Calot’s triangle due to acutely inflamed, edematous gallbladder or adhesions in 11 (2.2%) and excessive uncontrolled bleeding interfering with clear visibility in nine (1.8%) cases (Table 2). 

**TABLE 2:** Peroperative data of the patients

Characteristics	Year					Total (n=492) n(%)
	2011 (n=84) n(%)	2012 (n=92) n(%)	2013 (n=6) n(%)	2014 (n=98) n(%)	2015 (= 112) n(%)	
Conversion rate	7(8.3%)	4(4.3%)	6(5.7%)	3(3%)	4(3.6%)	24(4.9%)
Operative time (min)						
<60	22(26.2%)	15(16.3%)	27(25.5%)	22(22.4%)	31(27.7%)	117(23.8%)
60-89	53(63.1%)	73(79.3%)	70(66.0%)	75(76.5%)	80(71.4%)	351(71.3%)
≥90	8(9.5%)	5(5.4%)	7(6.6%)	2(2.0%)	2(1.8%)	24(4.9%)
Drainage	22(26.2%)	19(20.7%)	29(27.4%)	26(26.5%)	32(28.6%)	128(26%)
Hospital stay mean±SD (range) in days	3.1±2.1(1-11)	2.5±1(2-7)	2.8±1.8(2-11)	2.4±1.2(1-7)	2.4±1.2(1-7)	2.6±1.5(1-11)

In spite of the fact that 43 (8.7%) patients were ASA III, only 11 (2.2%) were kept in ICU for 2-4 days due to postoperative atrial fibrillation (n=5), bradycardia (n=4) and hypertension (n=2). 

Four patients were re-explored due to postoperative bleeding, they developed pallor, hypotension and dropped hematocrit values. Laparotomy was performed, the source of bleeding was cystic artery stump leakage in three cases and bleeding from the port site in the fourth case; bleeding control was performed by sutures (Table 3).

**TABLE 3:** Postoperative complications

Characteristics	Year					Total (n=492) n(%)
	2011 (n=84) n(%)	2012 (n=92) n(%)	2013 (n= 106) n(%)	2014(n=98) n(%)	2015 (n= 112) n (%)	
Postoperative bleeding	0(0%)	0(0%)	2(1.9%)	0(0%)	2(1.8%)	4(0.8%)
Bile leak	4(4.8%)	0(0%)	3(2.8%)	4(4.1%)	1(0.9%)	12(2.4%)
Wound infection	6(7.1%)	2(2.2%)	5(4.7%)	4(4.1%)	4(3.6%)	21(4.3%)
Collection in Morrison’s pouch	2(2.4%)	1(1.1%)	3(2.8%)	0(0%)	2(1.8%)	8(1.6%)
Port site hernia	3(3.6%)	2(2.2%)	2(1.9%)	0(0%)	2(1.8%)	9(1.8%)
Total cases with complications	15(17.9%)	5(5.4%)	15(14.2%)	8(8.2%)	11(9.8%)	54(11%)

There were 12 (2.4%) cases of biliary leakage. Seven (1.4%) proved to be due to partial tear in common bile duct, five of them were associated with common bile duct stones that were missed during the preoperative workup, and were referred to specialized center for ERCP and stone extraction, while the remaining two cases were successfully managed conservatively and biliary leakage stopped on the 10^th^ postoperative day. Regarding the other five cases, they were due to slipped cystic duct clipping and were successfully treated conservatively, biliary leakage stopped on the 7^th^ postoperative day in four cases, and on the 8^th^ postoperative day in the 5^th^ case (Table 3). There were no cases of bowel injury, major blood vessels injury or spilled gallstones. 

The mean hospital stay was (2.6±1.5 days) (1-11).

Twenty one (4.3%) patients developed wound infection that was treated by drainage and antibiotics (Table 3).

There was postoperative collection in Morrison’s pouch in eight cases (1.6%) diagnosed in the 7^th^ postoperative day during their follow up, they were readmitted and were managed by US guided tube drainage and antibiotics.

Port site hernia was detected in nine (1.8%) patients, and mesh repair was performed electively (Table 3).

During the period of follow up no patient developed postoperative bile duct stricture. There was no mortality recorded in this series.

## DISCUSSION

Gallstone disease is a global health problem. The incidence is 10-20% of the whole adult population, LC has now replaced open cholecystectomy as the first choice of treatment for gallstones, LC is performed in over 90% of elective cholecystectomies and 70% of emergency cholecystectomies making LC one of the most frequently performed operations in the world^13^.

Predictive factors of conversion to open cholecystectomy include male gender, previous abdominal surgery, acute cholecystitis, dense adhesions and fibrosis in Callot triangle, anatomical variations, advanced age, comorbidity, obesity, suspicion of common bile duct stones, jaundice, and decreased surgeon experience^5^.

In the literature, the rate of conversion from LC to open cholecystectomy varies from 2.6 to 7.7%^2^ ^,^ ^21^. Conversion results in a significant change in the outcome of the patients due to higher incidence of postoperative complications and longer hospital stay. In this study, the conversion rate was 4.9%, the main causes being anatomical variations, difficult dissection in Calot’s triangle and uncontrolled bleeding. 

The reported incidence of uncontrollable bleeding in LC can be up to 2% (0.03-10%). Operative bleeding results from major vascular injuries, liver injury, rough dissection at Callot’s triangle, or slipped clip from the cystic artery. The incidence of major vascular injuries is 0.03-0.06%. Major vascular injuries are the second most common cause of death in patients undergoing LC after anesthesia related complications^12^.

In this study, were encountered nine (1.8%) cases of uncontrollable intraoperative bleeding which nictitates conversion to open cholecystectomy, and four cases of postoperative bleeding which were managed successfully by laparotomy.

Abdominal drain was placed in patients who had intra-operative blood loss more than 100 ml or had biliary contamination from the gall bladder due to incidental perforation during its dissection.

Biliary injuries continue to be a significant problem following LC; most studies showed an increase in the incidence of these injuries. With the advent of laparoscopy, the rate of serious bile duct injuries after cholecystectomy increased up to 0.8%, whilst the one related to the open route remained between 0.2-0.3%^19^. The intraoperative detection of bile leak is around 40%, resulted mainly from failure to define the anatomy of Calot’s triangle. Endoscopic interventions have essentially replaced surgery as first line treatment for most of the biliary injuries following LC^4^. In this study were appointed seven cases of bile duct injuries, five of them managed by ERCP and the other two conservatively. 

Surgical site infection is significantly lower after laparoscopic surgery compared to open surgery and patients treated with laparoscopy were 72% less likely to experience an surgical site infection^11^ ^,^ ^17^. Were encountered 21 cases of wound infection (4.3%), which is consistent with the incidence in the literature. 

Port site hernia is considered as a rare complication after LC; however, there is a wide range of incidence reported in the literature between 0.14% and 22%^6^. It can lead to serious complications like irreducibility, intestinal obstruction, strangulation and perforation. In this study it was detected in nine (1.8%) patients who were managed by mesh repair.

## CONCLUSIONS

LC has proven to be a safe and effective procedure for the treatment of symptomatic gallstones at a secondary level of hospital care. It has several advantages compared with open cholecystectomy; however, when there is a major complication a multidisciplinary approach should be performed at a tertiary hospital.
